# More than meets the eye: Augmented reality in surgical oncology

**DOI:** 10.1002/jso.27790

**Published:** 2024-08-19

**Authors:** Kavita Prasad, Carly Fassler, Alexis Miller, Marina Aweeda, Sumit Pruthi, Joseph C. Fusco, Bruce Daniel, Michael Miga, Jie Ying Wu, Michael C. Topf

**Affiliations:** 1 Department of Otolaryngology-Head & Neck Surgery, Beth Israel Deaconess Medical Center, Boston, Massachusetts, USA; 2 Department of Otolaryngology-Head & Neck Surgery, Vanderbilt University Medical Center, Nashville, Tennessee, USA; 3 Department of Otolaryngology-Head & Neck Surgery, University of Alabama at Birmingham, Birmingham, Alabama, USA; 4 Department of Radiology, Vanderbilt University Medical Center, Nashville, Tennessee, USA; 5 Department of Pediatric Surgery, Vanderbilt University Medical Center, Nashville, Tennessee, USA; 6 Department of Radiology, Stanford Health Care, Palo Alto, California, USA; 7 Department of Biomedical Engineering, Vanderbilt University, Nashville, Tennessee, USA; 8 Department of Computer Science, Vanderbilt University, Nashville, Tennessee, USA

**Keywords:** augmented reality, cancer, surgical oncology, surgical technology

## Abstract

**Background and Objectives:**

In the field of surgical oncology, there has been a desire for innovative techniques to improve tumor visualization, resection, and patient outcomes. Augmented reality (AR) technology superimposes digital content onto the real-world environment, enhancing the user’s experience by blending digital and physical elements. A thorough examination of AR technology in surgical oncology has yet to be performed.

**Methods:**

A scoping review of intraoperative AR in surgical oncology was conducted according to the guidelines and recommendations of The Preferred Reporting Items for Systematic Review and Meta-analyzes Extension for Scoping Reviews (PRISMA-ScR) framework. All original articles examining the use of intraoperative AR during surgical management of cancer were included. Exclusion criteria included virtual reality applications only, preoperative use only, fluorescence, AR not specific to surgical oncology, and study design (reviews, commentaries, abstracts).

**Results:**

A total of 2735 articles were identified of which 83 were included. Most studies (52) were performed on animals or phantom models, while the remaining included patients. A total of 1112 intraoperative AR surgical cases were performed across the studies. The most common anatomic site was brain (20 articles), followed by liver (16), renal (9), and head and neck (8). AR was most often used for intraoperative navigation or anatomic visualization of tumors or critical structures but was also used to identify osteotomy or craniotomy planes.

**Conclusions:**

AR technology has been applied across the field of surgical oncology to aid in localization and resection of tumors.

## 1 | INTRODUCTION

Over the past 30 years, virtual and augmented reality (AR) technologies have emerged as rapidly growing areas of research and development. The distinguishing factor between virtual reality (VR) and AR is that the former completely immerses the user in a computer-generated world, while the latter creates a composite view of the real world by overlaying digital images onto the user’s view. A variety of industries, from the military to medicine, have found applications for these technologies in education, simulation, and beyond.

Unlike VR, AR has more potential to be used intraoperatively by allowing the surgeon to import two-dimensional (2D) or three-dimensional (3D) holographic models into the operating room environment while still visualizing the surgical field. Intraoperative AR has become an established avenue of investigation in a variety of surgical subspecialties. In orthopedics, this technology has been used for presurgical planning and intraoperative guidance of screw placement.^1,2^ In neurosurgery, 3D holograms of preoperative imaging studies have increasingly represented a new modality for surgical navigation.^3–5^ The technology has been evaluated in its ability to reduce positive margin rates in a number of subspecialties including the head and neck and genitourinary surgical oncology.^6,7^

In the field of surgical oncology, there has been a desire for innovations and novel techniques to improve tumor visualization, resection, and overall patient outcomes. Positive margin rates remain high for many solid malignancies and subsequently impact patient prognosis.^8^ A thorough examination of how AR is being applied across the surgical oncology field has yet to be performed. We aim to conduct the first scoping review of AR applications across all surgical oncology subspecialties.

## 2 | METHODS

We conducted a review of AR in surgical oncology according to the guidelines and recommendations of The Preferred Reporting Items for Systematic Review and Meta-analyzes Extension for Scoping Reviews (PRISMA-ScR) framework (Appendix S1).^9^ A scoping review was deemed preferred given the novelty of the technology lending most articles to be feasibility or accuracy studies in nature.

### 2.1 | Search strategy

A comprehensive literature search of the PubMed, Embase, and Web of Science databases was conducted. The following search terms were used: “mixed reality” OR “virtual reality” OR “augmented reality” OR “holography” OR “hologram” AND “surgery” OR “surgical” AND “oncology” OR “cancer” OR “tumor” OR “neoplasm.” Complete search strategies for each database are presented in Appendix S2.

### 2.2 | Inclusion criteria

We sought to identify all original articles examining the use of intraoperative AR during surgical management of cancer. Both pre-clinical and clinical works were included. Inclusion criteria were any studies reporting the use of AR in any oncologic surgical case.

### 2.3 | Exclusion criteria

Articles were excluded for the following reasons: VR applications only, preoperative use only (no intraoperative use of AR), fluorescence-guidance deemed AR, not specific to surgical oncology or study design (reviews, commentaries, conference abstracts without a corresponding full text).

### 2.4 | Study selection

Two researchers (K.P. and A.M.) independently reviewed all the studies (title, abstract, and full text) that met the inclusion criteria. All relevant data were extracted. Conflicts between reviewers were resolved through discussion.

## 3 | RESULTS

### 3.1 | Eligible studies

**FIGURE 1:**
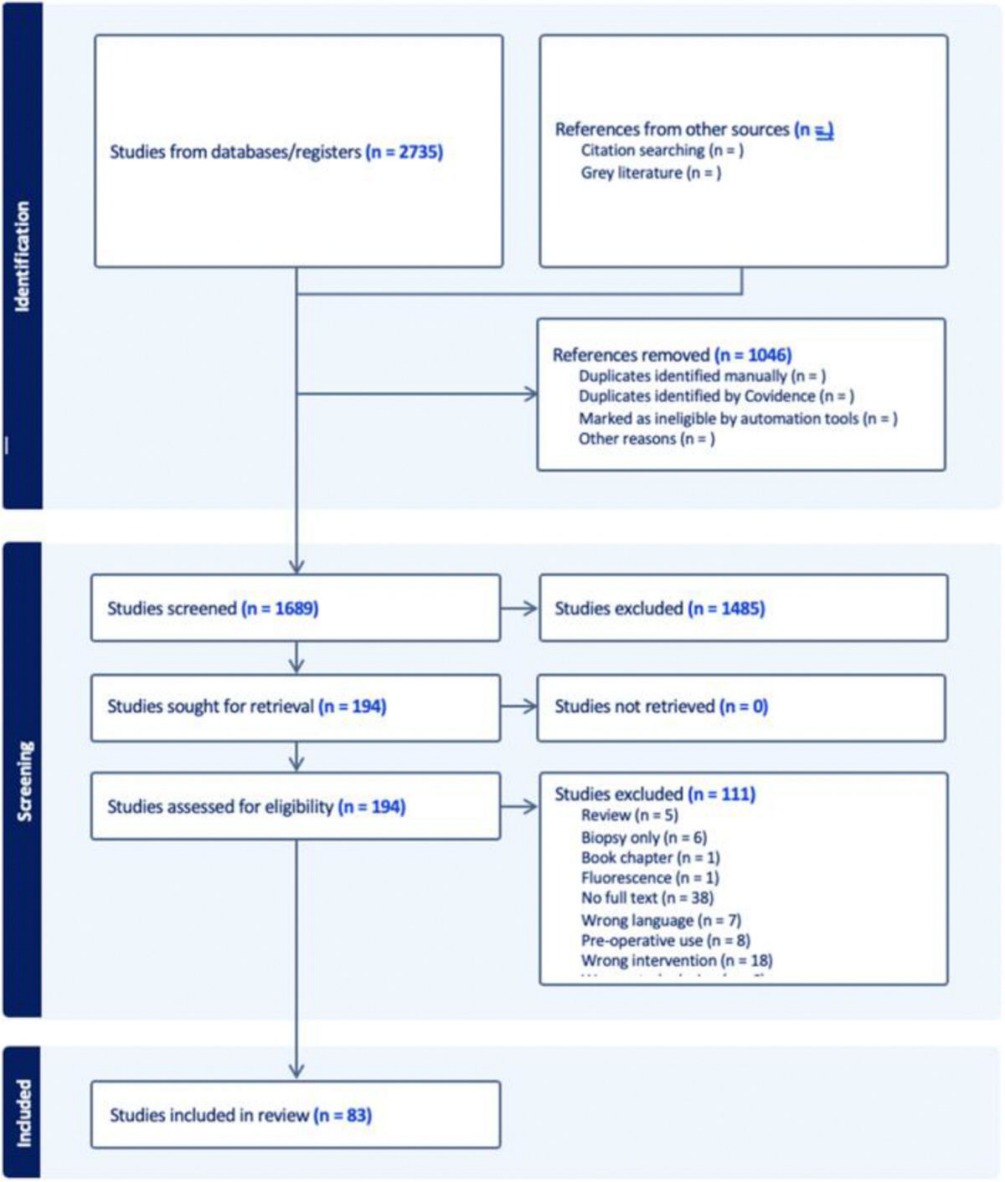
Mixed reality scoping review flow diagram.

The search strategy yielded 2735 articles (Figure 1). The review tool, Covidence (Melbourne, Australia) was utilized to conduct the formal review. A total of 1046 duplicates were removed. Most studies were excluded due to duplication, preoperative use only, or use of VR rather than AR.

**FIGURE 2:**
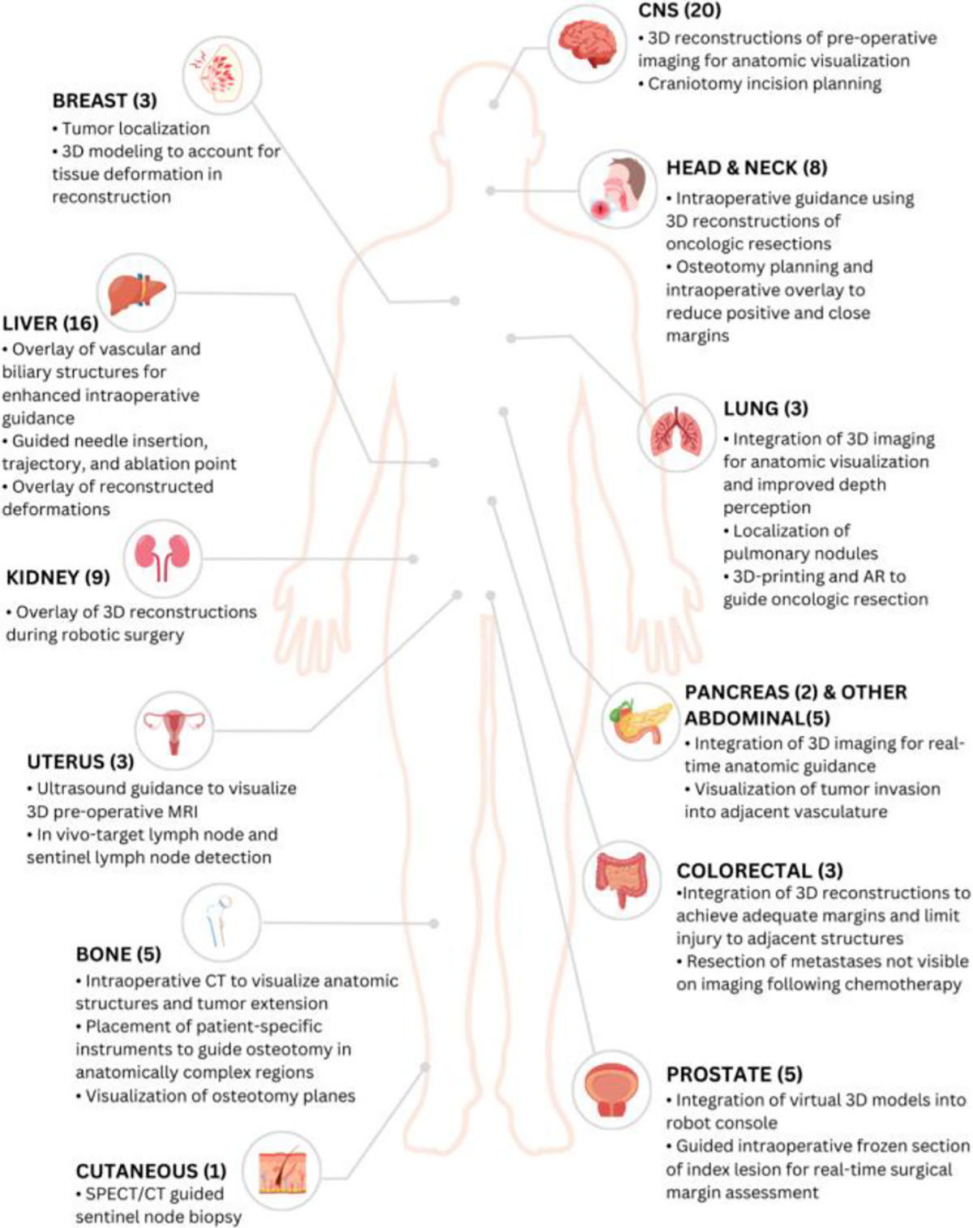
Augmented reality (AR) use-cases across organ systems.

A total of 83 papers were extracted and grouped by anatomic subsite (Figure 2). Results are presented based on primary tumor location: brain, liver, kidney, head and neck, bone, prostate, breast, colorectal, lung, uterus, pancreas, other abdominal (gallbladder, esophagus, adrenal), cutaneous. Each section is further divided based on how AR was used, i.e. navigation, and margin identification. Studies that investigated and reported accuracy of the AR intervention are detailed in Table 1.

#### I. BRAIN (20)

Twenty articles described the use of AR in surgical management of brain cancer.^4,5,10–27^

##### i. Navigation/anatomic visualization

Most studies (11) utilized AR for intraoperative guidance.^4,5,10–13,15,16,18,19,25^ Preoperative imaging studies were overlaid onto the surgical field within the heads-up display of the microscope (4)^4,13,16,25^ or within the Microsoft HoloLens (2),^5,19^ a tablet PC (2),^11,15^ a projector (2),^12,18^ or a smartphone (1).^10^ Most studies segmented vasculature and tumor boundaries within the 3D reconstructions. Bernard et al.^25^ created AR-assisted road-maps during periventricular brain surgery to improve visualization of periventricular structures during tumor removal.

AR was also used during transsphenoidal surgeries for enhanced anatomic visualization and navigation.^20,23,24,26^ 3D reconstructions of preoperative imaging studies were integrated into the heads-up display of either the operating microscope or endoscope. Carl et al.^23^ segmented the imaging studies to highlight critical structures, including the tumor, chiasm, and optic nerve and tracts. Carl et al.^23^ and Bopp et al.^24^ used intraoperative CT (iCT) to help accurately align the AR navigation. Carl et al.^23^ found a mean AR registration error of 0.83 ± 0.44 mm, while Bopp et al.^24^ found a target-registration of 0.76± 0.33 mm. Zeiger et al.^26^ developed a new mixed reality system for anatomic visualization and guidance during endoscopic anterior skull base and complex paranasal procedures.

##### ii. Craniotomy planning

Six studies used AR to improve the accuracy of craniotomy incisions.^14,15,17,21,22,27^ Preoperative craniotomy planning was conducted using 3D models of imaging studies. Schwam et al.^27^ used AR for craniotomy planning and precision in lateral skull base surgery for cerebellopontine angle tumors. At the same time, Montemmuro et al.^17^ applied a similar technique for parasagittal convexity enplaque meningiomas. The predetermined incision boundaries and trajectory were overlaid onto patients’ scalps using either a microscope,^27^ smartphone,^14^ HoloLens,^21^ 3D visor (SonyHMZ-T2),^22^ or VOSTARS head mount display.^17^

#### II. LIVER (16)

Sixteen articles discussed applications of AR in liver cancer resection.^28–43^

##### i. Navigation/anatomic visualization

Most studies evaluated intraoperative navigation using AR technology. Anatomic guidance was overlaid onto the patient and visualized using either the HoloLens or the laparoscope console. Shi et al.^31^ proposed a novel internal/external correlation model to estimate and adjust for the motion of the internal structures. They performed two simulated cases and found an average tumor estimation error of the tumor and vessels were 2.18 and 2.79 mm.

##### ii. Ablation

AR was also used to guide need insertion in radiofrequency ablation procedures.^37–42^ Two studies superimposed 3D reconstructions of intraoperative ultrasound images to guide liver tumor puncture.^39,42^ The remaining studies used either the HoloLens or external projector to visualize preoperative images and plans on the patient’s body to guide needle insertion, trajectory, and ablation point. Li and colleagues^40,41^ used the HoloLens in conjunction with an optical tracking system, NDI Polaris, for improved position-tracking and calibration.

##### iii. Deformation modeling

Given surgeons’ documented difficulty with deformation during hepatic surgery, three studies attempted to model and overlay deformed reconstructions of the preoperative imaging studies.^30,34–36^ All three studies developed their mathematical formulas to model volume-surface deformations. Two studies demonstrated success in human patients; one achieved system registration and calibration in an average of 113 s,^34^ and the other successfully used deformable models in AR to locate tumors in 88% of cases.^35^ Additionally, one study demonstrated a surface registration error of less than 6 mm in identifying tumor positions in porcine liver models.^36^

#### III. KIDNEY (9)

Nine studies evaluated the use of intraoperative AR in the treatment of renal cancer.^44–52^

##### I. Navigation/anatomic visualization

The most common application of AR was for navigation during partial nephrectomy. Holographic 3D reconstructions of imaging studies were either integrated into the laparoscope console during robotic-assisted partial nephrectomy^45–49^ or projected above the patient^50,51^ in the operating room using the HoloLens. One study used a combination of preoperative CT scans and intraoperative images to accurately align the holographic model with the patient’s anatomy. It achieved a target registration error of 0.5 mm (range: 0.2–0.7 mm).^47^

##### II. Radiofrequency

One study fused intraoperative ultrasound with preoperative CT and MRI scans with the laparoscopic view to guide needle placement for radiofrequency treatment of a renal tumor.^44^ The authors designed a color-coded zonal navigation system: red for the tumor, yellow for the 0–5-mm margin, green for the 5–10-mm margin, and blue for more than 10 mm. This allowed the surgeon to visualize both the image of the tumor and the surrounding safety zones, providing them with a 3D visual “road map.”

#### IV. HEAD & NECK (8)

##### i. Nine studies utilized AR in the surgical management of head and neck tumors.^53–60^ *Navigation/Anatomic visualization*

Most studies superimposed 3D reconstructions of preoperative CTs or MRIs for improved visualization and guidance in the resection of maxillary,^56^ parotid,^57^ base of tongue,^58^ external ear,^59^ and laryngeal tumors.^60^ Common AR modalities included the HoloLens, the laparoscope, and the endoscope. Scherl et al.^57^ evaluated the registration accuracy of MRI DICOM files onto the patient during parotid surgery. They found a mean target registration accuracy of 1.3 cm with significant differences seen in position error of registration between central and peripheral structures (*p* = 0.0059). Prasad et al.^6^ evaluated the feasibility of AR technology to guide head and neck cancer re-resections after an initial positive margin. In their cadaveric study, they realigned 3D specimen holograms of tumor specimens back into the surgical defect with a mean relocation error of 4 mm.

##### ii. Osteotomy planning

AR was also utilized in virtual preoperative planning and intraoperative overlay of osteotomy boundaries and trajectories for the removal of maxillary and mandibular tumors. Tang et al.^53,54^ found mean deviations of maxillary and mandibular osteotomy lines were approximate (1.60 ± 0.93 mm and. 1.86 ± 0.93 mm, respectively).

##### iii. Margins

**FIGURE 3:**
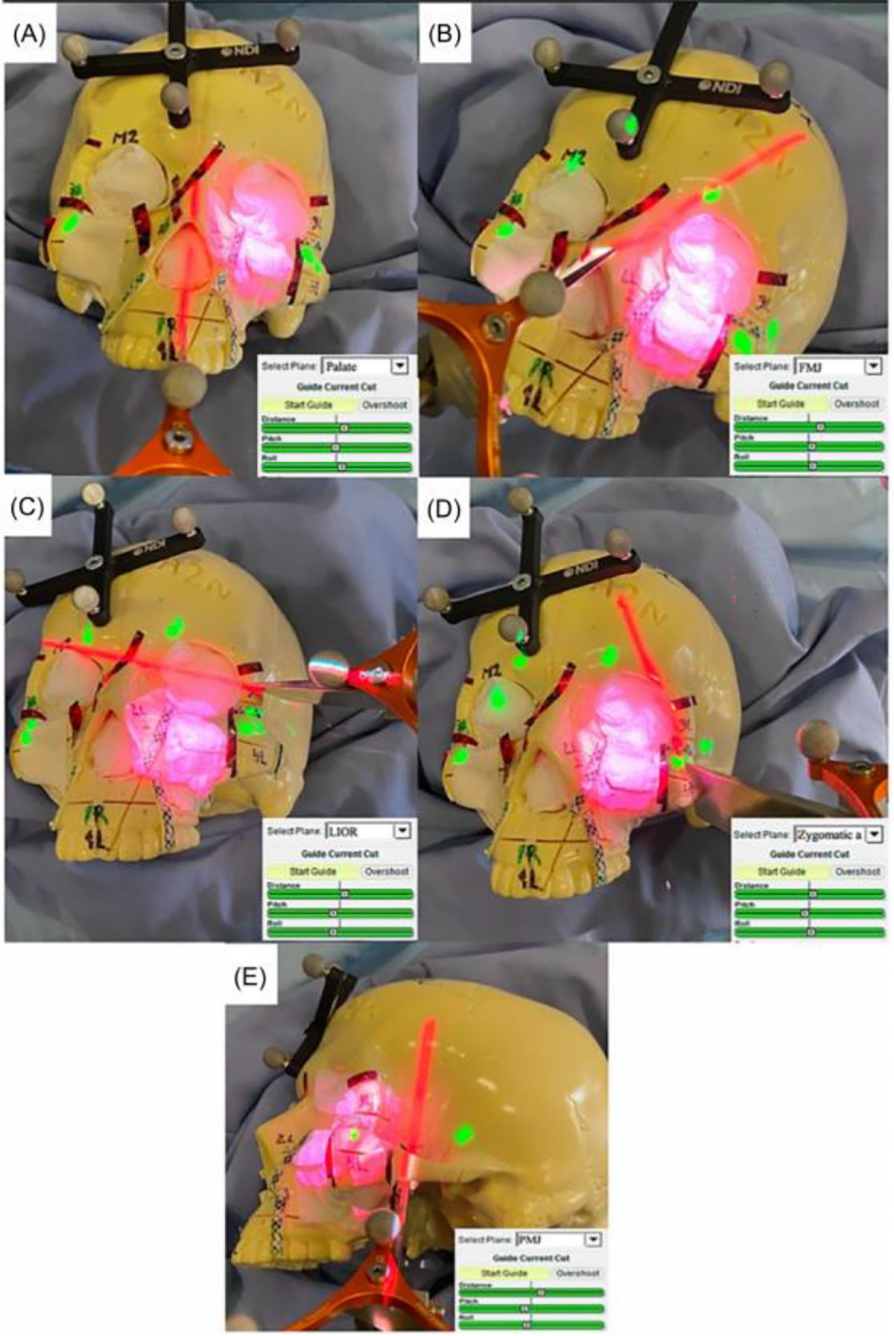
Example of the different AR projected osteotomies required to resect a left-sided maxillary tumor in sequence, with zoom-ins into the distance pitch and roll cutting parameters projected onto the surgical field; (A) Palate, (B) Fronto-maxillary junction, (C) Lower inferior orbital rim, (D) Zygomatic arch, and (E) Pterygomaxillary junction. This figure was reproduced from Chan et al.^54^ AR, augmented reality.

Two studies investigated the impact of AR on intratumoral cut-through.^54,55^ In their preclinical study, Chan and colleagues^54^ found positive and close margins were lower for the AR-assisted osteotomies for maxillary tumors (0.0% vs. 1.9%, *p* < 0.0001 and 0.8% vs. 7.9%, *p* < 0.0001) (Figure 3). In the management of sinonasal malignancies, intra-tumoral cuts were observed in 20.7% unguided versus 9.4% of AR alone simulations.^55^

#### V. BONE (5)

Five articles used AR in the treatment of bone and spine tumors.^61–65^

##### i. Navigation/anatomic visualization

Carl et al.^65^ segmented and overlaid iCT images to visualize the vertebra and tumor extension in spinal tumor surgery. Moreta-Martinez et al.^61^ used a smartphone application to display and align 3D models of thorax, femur, hip, and tibial tumors onto the respective patient intraoperatively, establishing a mean registration accuracy of 2.80 ± 0.98 mm. Cho et al.^63^ investigated the accuracy of tablet-PC based AR navigation application, performing 246 resections on 123 animal femur models and found a mean error of 1.71 compared to 2.64 for conventional resection (*p* < 0.05).

##### ii. Osteotomy planes

Two studies used tablet PC or smartphone-based AR applications to superimpose resection and osteotomy planes.^62,64^ Choi and colleagues^62^ projected desired surgical resection margins with real-time tracking and adjustments onto a pelvic tumor model. When the target resection margin was 10 mm, the measured resection margin using the conventional method was 7.11 ± 4.30 mm, whereas with the AR protocol, it was 9.85± 1.02 mm. Garcia-Sevilla discussed some surgeons’ difficulty with placing patient-specific instruments (PSI) to guide osteotomy in anatomically complex regions such as the pelvis. They found the use of both a smartphone-based or HoloLens AR application to guide the placement of PSI in pelvis tumor removal.

#### VI. PROSTATE (5)

Five articles discussed the application of AR to the surgical treatment of prostate cancer.^7,66–69^

##### i. Navigation/Anatomic visualization

Four studies investigated the integration of virtual 3D models into the da Vinci surgical console during robotic-assisted prostatectomy (RARP). Puliatti et al.^68^ combined MRI imaging and a statistical tool for extracapsular extension. Porpiglia and colleagues^69^ investigated a hyper-accuracy 3D model based on multiparametric MRI to guide selective biopsies to confirm suspected extracapsular extension at the level of the neurovascular bundle.

##### ii. Margins

Checcucci and colleagues^7^ evaluated the impact of AR on positive surgical margins and found that AR-guided RARP led to a lower positive margin rate (25.0% vs. 35.1%, *p* = 0.01). Bianchi and team^66^ used AR to guide intraoperative frozen section of the index lesion for real-time surgical margin assessment during nerve-sparing RARP. They determined that positive surgical margin rates at the level of the index lesion were significant lower with AR guidance (5% vs. 20%, *p* = 0.01).

#### VII. BREAST (4)

Four articles discussed the use of intraoperative AR for breast cancer surgery.^70–72^

##### i. Navigation/anatomic visualization

Given the well-known discrepancy in breast deformation and tumor localization between prone and supine patient positioning, all studies evaluate the use of AR technology for tumor localization. Perkins et al.^70^ evaluated the application of AR to improve the accuracy of lumpectomy for early-stage breast cancer. They used the Microsoft HoloLens to overlay holograms of preoperative MRI onto the patient in the operating room. They used the Microsoft HoloLens to overlay holograms of preoperative MRI onto the patient in the operating room and found a signed distance error that ranged between −6.1 and 7.1 mm in the up-down dimension and between −3.3 and 2.2 mm in the left-right dimension. Gouveia et al.^71^ combined preoperative image scans with a 3D surface scan taken of the patient in the operating room to account for tissue deformation and projected this onto the patient using the HoloLens. Sato and colleagues^72^ reconstructed intraoperative ultrasonic images into a 3D model and super-imposed this onto the patient using an external projector. Pallone et al.^73^ evaluated whether superimposed images of supine MRI studies could be aid in tumor localization of nonpalpable tumors during breast conserving surgery. They determined that average distance from the palpated and supine MRI image-defined tumor edge was 7.2 mm, though this improved over time, with the last seven patients averaging a 4 mm away.

#### VIII. COLORECTAL (3)

Three studies used AR assistance in the treatment of colorectal cancer.^74–76^

##### i. Navigation/anatomic visualization

3D reconstructions of intraoperative ultrasound were used to achieve adequate resection margins for lower rectal cancer. Ryu et al.^76^ performed a total of 2 ileocecal resections, 6 right hemicolectomies, 1 partial colectomy, 4 lateral lymph node dissections, and 1 para-aortic lymph node dissection while visualizing 3D reconstructions of CT images hovering above the patient in the operating room through the HoloLens. They used the NASA Task Load Index to quantify the usefulness and efficacy of holographic guidance. They found that surgeons felt this system had a low burden on performance and mental demand but a higher score on physical demand and frustration. Shen and colleagues^75^ combined intraoperative ultrasound with the standard endoscope system to guide tumor resection with adequate margins for lower rectal cancer. They determined a mean error of 0.9 mm for their transrectal ultrasound calibration. Finally, Tokunga et al.^74^ used AR during transanal total mesorectal excision (TaTME) to limit urethral injury, a critical complication, by visualizing 3D reconstructions of CT images using the HoloLens.

#### IX. LUNG (3)

Three studies applied AR in surgical management of lung cancer.^77–79^

##### i. Navigation/anatomic visualization

Holographic 3D projections of CT scans were visualized in the HoloLens and aligned to the patient for localization of pulmonary nodules. Additionally, preoperative imaging scans were integrated into the laparoscopic display for anatomic visualization and depth perception during complex thoracoscopic surgery.^78^ Peng et al.^77^ performed AR-assisted localization of solitary pulmonary nodules for precise sublobar resection. They found the average distance between the puncture point and the expected point to be 8.74 ± 5.07 mm, and the difference between the actual and expected depths was 9.42 ± 7.95 mm. Another study investigated the combination of 3D-printing and AR to guide segmentectomies and sub segmentectomies.^79^ They found that the AR method led to shorter operating times and less intraoperative blood loss than the conventional laparoscopic guidance.

#### X. UTERUS (3)

Three studies evaluated the use of AR in gynecologic surgery. All three were preclinical.^80–82^

##### i. Navigation/anatomic visualization

One study aimed to improve upon the standard-of-care ultra-sound guidance during myomectomy by using the HoloLens to hover 3D preoperative MRI scans above the patient intraoperatively.^80^ This study qualitatively assessed the usefulness of the renderings for fibroid localization using an HMD compared to the same visualization on a 2D monitor.

##### ii. Lymph node detection

**FIGURE 4:**
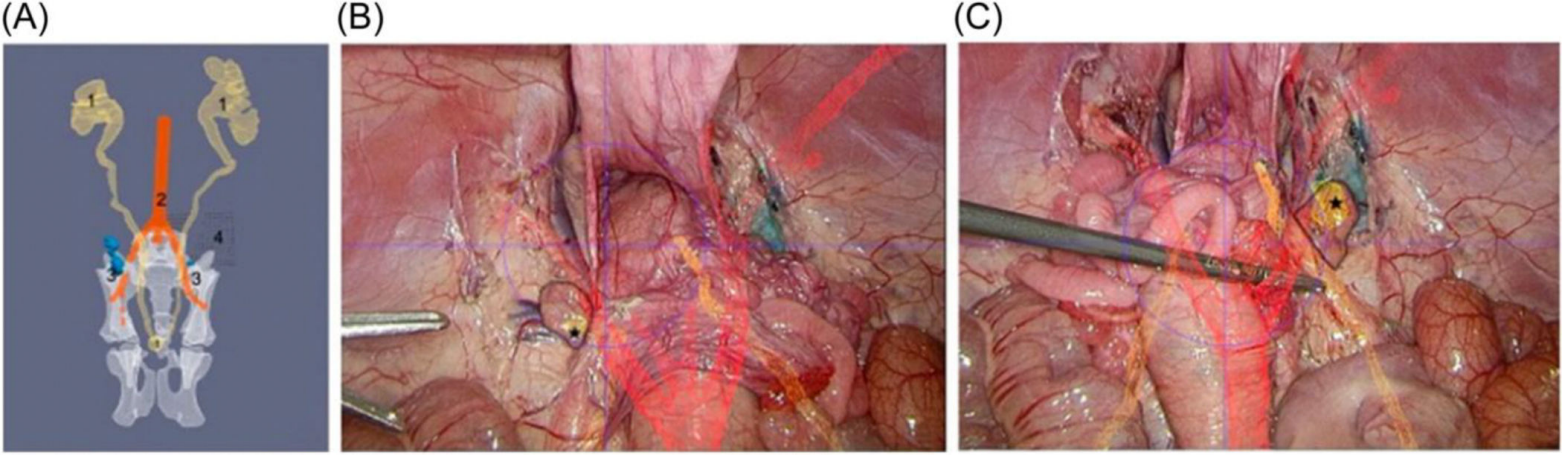
Sequence of segmentation and AR overlay. Segmentation of CT images with 3D reconstruction of pelvic structures [(A) urinary system (1) with ureters and intravesical balloon, arteries (2), lymph nodes (3), and calibration checkerboard (4)] enabling the augmented reality application. Laparoscopic view of the pelvic anatomy enhanced by real-time AR overlay of the SLN and anatomical landmarks (B,C) with the SLN marked by a black asterisk on the left (B) and right (C) side. This figure was reproduced from Lecointre et al.^81^ AR, augmented reality.

Lecoiontre et al.^81^ conducted two studies evaluating AR for in vivo-target lymph node and sentinel lymph node (SLN) detection for endometrial cancer patients. In the first study, they overlaid a combination of preoperative SPECT and iCT imaging onto the body with a coverage rate of 99.48% on the left SLN and 99.42% on the right SLN (Figure 4).^81^ The second study highlighted the development of an AR-based robotic assistance system that performed real-time multimodal and temporal fusion of laparoscopic images with preoperative CT images and found the accuracy of the CT overlay was >90%, with overflow rates <6%^80^

#### PANCREAS (2)

Two studies evaluated AR during pancreaticoduodenectomy (PD).^83,84^

##### i. Navigation/anatomic visualization

**FIGURE 5:**
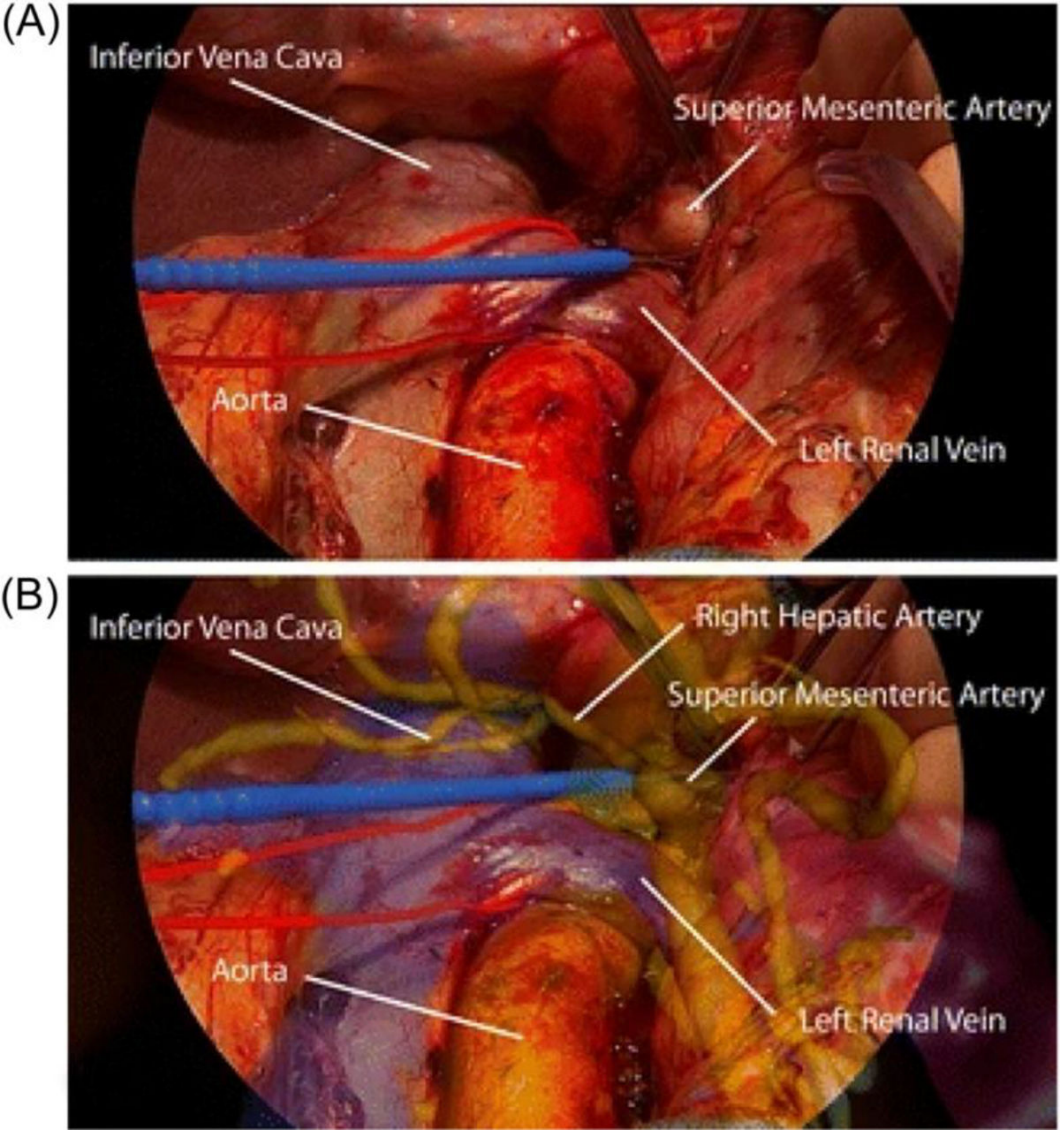
(A) Dissection of the superior mesenteric artery (SMA) at its origin. (B) Augmented reality of the dissection of the superior mesenteric artery at its origin. This figure was reproduced from Marzano et al.^84^

3D CT images were integrated onto the Exoscope display for real-time anatomic guidance during PD with an artery-first approach for ampullary adenocarcinoma (Figure 5).^84^ During PD with superior mesenteric vein resection, pancreatic head tumor invasion into the SMV was visualized by aligning 3D preoperative scans onto the patient using a smartphone application.^83^

##### XI. OTHER ABDOMINAL

Five studies examined AR during surgical treatment of different intra-abdominal malignancies.^85–89^

##### I. Navigation/anatomic visualization

Video-based in situ 3D anatomical modeling on an iPad was used during open hilar cholangiocarcinoma resection with hemihepatectomy.^86^ Patient CT and MRCP data were used to reconstruct computer-generated, optimized images by aligning blood vessel bifurcations in the 3D images with corresponding structures in a 3D-printed model of the tumor, involving biliary ducts and hepatic vasculature. 3D images were overlaid onto video images of the surgical field for preoperative planning and intraoperative AR-assisted navigation.

The HoloLens was used to display 3D holograms during thoracoscopic esophagectomy and improve spatial understanding of the anatomical variation of the right vertebral artery.^87^

Eight cases of single-incision laparoscopic adrenalectomy for cortical adenoma were performed with AR guidance using 3D reconstructions of preoperative imaging.^89^

The AR software loads the 3D model, connects to the laparoscope, and in real time merges the two views together. Operative times were found to be shorter in the AR-guided surgery group compared to the standard surgery group (*p* = 0.005).

#### XII. CUTANEOUS

##### i. Lymph node biopsy

Van den Berg and colleagues^90^ evaluated the feasibility of SPECT/CT based AR-navigation for sentinel node biopsy in patients with melanoma or Merkel cell carcinoma in the lower extremity. This gave surgeons an interactive roadmap to navigate toward the sentinel node with an average navigation error of 8 mm sagitally and 8.5 mm coronally.

## 4 | DISCUSSION

In this review, we present 83 papers detailing the use of AR technology in oncologic surgery. Fifty-two studies were performed on animals or phantom models, while the remaining were performed on patients. A total of 1112 cases with intraoperative AR were performed across the studies. Twenty studies overlaid imaging on the external skin of the body for percutaneous procedures, 58 investigated AR holograms aligned directly on internal organs, and the 5 remaining studies simply projected the 3D model over the patient’s body without alignment. Fourteen unique organs are represented, with the most common tumor site being brain, followed by liver, and head and neck.

AR technology was most often used for intraoperative navigation and visualization of anatomic structures. The technology was commonly integrated into the laparoscope or endoscope console to improve spatial understanding of critical structures such as the tumor and surrounding vessels. Additionally, a few investigators examined the use of AR to reduce positive surgical margin rates. This was most prevalent in the prostate and head and neck literature, which happened to be the two anatomic subsites with the highest positive margin rates of all solid malignancies.^8^ Finally, some studies highlighted the ability of AR to improve the identification of SLNs based on 3D reconstructions of SPECT/CT imaging.

The application of AR technology intraoperatively may be easier on certain tissue types, such as bone. In surgical management of tumors that involve bone, whether it be in orthopedics, neurosurgery, or the head and neck, surgeons were able to use AR to plan and accurately visualize osteotomy and craniotomy planes. VR and AR were used preoperatively to plan and annotate imaging studies with the desired incision sites and trajectories. Given the rigidity of bone, plans were superimposed onto the patient with good accuracy using the HoloLens. For anatomic subsites that are more deformable, such as the liver and breast, surgeons previously described difficulty relocating the tumor intraoperatively due to tissue shifting and inconsistencies with the preoperative scans. For deformable soft tissue tumors, AR combined with mathematical modeling or ultrasound-based reconstruction to estimate deformation in real-time and improve guidance of the new location of the tumor shows early promise.^30,34–36^

This review highlights the consensus across the field of surgical oncology for improved intraoperative tools for tumor localization, precision surgery, and oncologic fidelity, with the goal of improving cancer patient outcomes. Several studies investigated the clinical outcomes of AR-guided surgery as compared to conventional guidance systems. Many cited shorter operative times and lower intraoperative blood loss with AR technology.^29^ For example, Zhang et al.^29^ found that patients who received AR-guided hepatectomy had significantly less operative bleeding (*p* = 0.002), lower delta Hb% (*p* = 0.039), lower blood transfusion rate (*p* < 0.001), and reduced postoperative hospital stay (*p* = 0.003) than those who received standard liver tumor resection. Furthermore, numerous studies found that AR-guided tumor resection had significantly lower positive margin rates than the current standard.^7,52,54,55,66,76^ In head and neck tumors, Chan et al.^54^ found that close margins were lower for the AR-assisted osteotomies for maxillary tumors (0.8% vs. 7.9%, *p* < 0.0001) (Figure 3). For sinonasal malignancies, positive margins were observed in 20.7% without AR guidance versus 9.4% with AR.^55^ In management of pelvic bone tumors, Choi and colleagues^62^ found that an AR protocol was more likely to achieve the adequate target margin of 10 mm, with a mean resection 9.85 ± 1.02 mm compared to 7.11 ± 4.30 mm with the conventional method. In prostate cancer resection, Checcucci and the team^7^ found the positive margin rate to be lower with AR guidance (25% vs. 35.1%, *p* = 0.01). Given the impact of a positive margin on patient outcomes across tumor types, the ability to reduce positive margin rates with AR is critical. Finally, precision surgery not only limits both under- and over-excision, it also enables patients to stay on their treatment timeline by preventing disruption of their adjuvant chemotherapeutic or radiation regimens.

As with any new surgical technology, the accuracy of the novel intervention must be evaluated. In a review investigating error reporting in image-guided surgery (IGS) systems in otolaryngology, Labadie and colleagues^91^ found that IGS systems most commonly report a mean accuracy of 2 mm or less. Another review article found that neuronavigation systems operate with a mean application accuracy of 1.5–5.4 mm.^92^

In our review of AR for surgical oncology, 32 studies reported the accuracy of their AR intervention, with hepatobiliary representing the greatest proportion of these studies (Table 1). As AR modality (i.e., HoloLens, exoscope, tablet) and outcomes reporting were not standardized, we could not compare or draw conclusions across studies. However, 28 studies established an accuracy of within 5 mm. Of the four remaining studies with accuracy errors of greater than 5, three of them were for liver cancer surgery. This may be due to the deformable nature of the liver, requiring mathematical modeling to account for these changes intraoperatively. A couple of studies investigated fiducial-based versus iCT-based registration systems and found iCT to be more accurate. Future studies must seek to evaluate the ideal modality and appropriate error range for each anatomic subsite.

Despite the exciting potential of this technology highlighted in this review, there are still significant limitations to the intraoperative use of AR in surgical oncology. The majority of these studies were feasibility in nature and did not disclose clinical or accuracy data. Additionally, few studies investigated the subjective experience and burden on the surgeon. Ryu and colleagues^76^ used the NASA Task Load Index and found that surgeons felt this system had a low burden on performance and mental demand, but higher score on physical demand and frustration. Further understanding of usability, efficacy, and perceived usefulness is essential to understand the adoptability of this technology. Future studies should investigate a surgeon’s perception of the realistic nature of the AR content and its representation in space. Finally, as AR is a relatively new, though rapidly evolving, technology, most studies developed their own software for use. Future development of software and applications will enable transferability.

**FIGURE 6:**
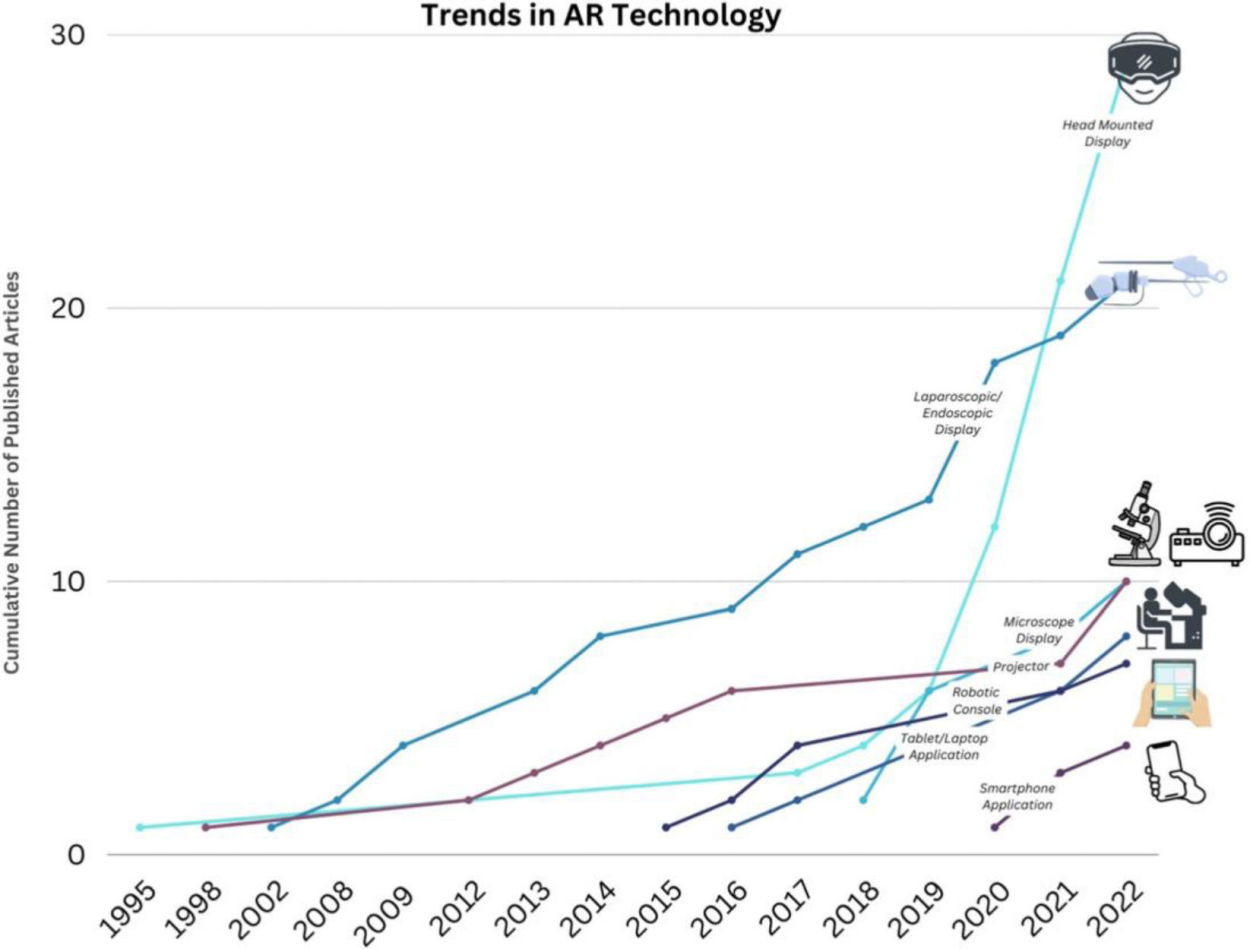
Trends in augmented reality (AR) technology use from 1995 to 2022.

Within the next decade, AR may become ubiquitous in operating rooms, even as simply a hands-free means of visualizing preoperative imaging studies during surgery. Figure 6 highlights the rapid adoption of this technology, rivaling that of conventional image guided surgery. Once there is definitive evidence, reproducible evidence on the accuracy and impact on patient outcomes, the use of AR in surgical oncology and other surgical subspecialties may become standard of care.

## 5 | CONCLUSIONS

In conclusion, the emergence of AR technology presents a promising avenue for enhancing surgical interventions in oncology. This scoping review, encompassing a comprehensive analysis of 83 studies representing 14 solid malignancies, underscores the diverse applications and rapid adoption of AR across various oncologic subspecialties. With a focus on intraoperative guidance, visualization, and margin identification, AR has demonstrated potential in improving surgical precision, reducing positive margin rates, and ultimately enhancing patient outcomes.

## Supplementary Material

Supporting Information

SUPPORTING INFORMATION

Additional supporting information can be found online in the Supporting Information section at the end of this article.

## Figures and Tables

**TABLE 1:** Accuracy of published augmented reality studies in surgical oncology.

Anatomic site	Accuracy	Comparison group
Breast	Error in up-down dimension (mean standard deviation) [range] −1.03.5 [−6.1, 7.1] mm Error in right-left dimension (mean standard deviation) [range] −0.21.3 [−3.3, 2.2] mm Error in up-down dimension (mean standard deviation) [range] −1.12.0 [−5.9, 2.3] mm Error in right-left dimension (mean standard deviation) [range] 0.11.2 [−2.2, 3.0] mm Margin tolerance [0.68, 5.74] mm Dice coefficient [0.56, 0.95”]	
Colorectal	0.8 mm (mean error)	
Gastrointestinal	3–5 mm (average accuracy)	
HN	1.60 ± 0.93 mm (maxillary osteotomies) 1.86 ± 0.93 mm (mandibular osteotomies)	
HN	13 mm (mean accuracy)	
HN	4 mm	
Liver	1.88 mm	
Liver	3.92 mm (Phantom)	
Liver	5.33 (animal model)	
Liver	2.04 (tumor)	
Liver	4.79 (vessel)	
Liver	12.4 (manual registration)	
Liver	4.3 (semi-automatic registration)	
Liver	2.45 mm	
Liver	11.1 mm	14.9 mm (conventional)
Liver	4.89 mm	9.02 mm (w/o AR)
Liver	3.52 mm	15.59 mm
Liver		
Lung	8.74 ± 5.07 mm	
Lung	9 mm	
Brain	1.70 ± 1.02 mm,	
Brain	0.8 ± 0.25 mm	
Brain	4.7 ± 2.3 mm	
Brain	0.82 mm + − 0.37	
Brain	0.3 mm	
Brain	2.33 ± 1.30 mm (fiducial-based) 0.83 ± 0.44 mm (iCT)	
Brain	0.76 ± 0.33 mm (iCT) 1.85 ± 1.02 mm (Landmark based)	
Bone	2.80 ± 0.98mm	
Bone	0.58 ± 0.22 mm	
Bone	1.71 mm	2.64 (conventional)
Bone	<5 mm	54.3 mm (conventional)
Bone	0.72 ± 0.24 mm	
Cutaneous	The average navigational error was 8.0 mm in the sagittal direction and 8.5 mm in the coronal direction	
Pancreas	2.93 mm	
Renal	mm	

## Data Availability

Data sharing is not applicable to this article as no new data were created or analyzed in this study.
